# Multidisciplinary management of Whipple’s disease complicated with rectal stenosis from multidisciplinary nursing management: A case study

**DOI:** 10.12669/pjms.42.9.16422

**Published:** 2026-09

**Authors:** Fang Ding, Lili Dong, Yahong He, Wenli Wu, Rong Xu

**Affiliations:** 1 Fang Ding, Department of Gastroenterology, Hangzhou Third People’s Hospital, Hangzhou, Zhejiang Province 310009, P. R. China; 2 Lili Dong, Department of Gastroenterology, Hangzhou Third People’s Hospital, Hangzhou, Zhejiang Province 310009, P. R. China; 3 Yahong He, Department of Gastroenterology, Hangzhou Third People’s Hospital, Hangzhou, Zhejiang Province 310009, P. R. China; 4 Wenli Wu, Department of Gastroenterology, Hangzhou Third People’s Hospital, Hangzhou, Zhejiang Province 310009, P. R. China; 5 Rong Xu, Department of Gastroenterology, Hangzhou Third People’s Hospital, Hangzhou, Zhejiang Province 310009, P. R. China

## Abstract

Whipple’s disease (WD) is a rare, chronic systemic disorder caused by Tropheryma whipplei (*T. whipplei*), a bacterium belonging to the Actinobacteria phylum. Clinical manifestations of WD include weight loss, diarrhea, and arthralgia. To date, no cases of rectal stenosis caused by *T. whipplei* infection have been reported in the literature. This paper presents a case of rectal stenosis associated with *T. whipplei* infection, evaluates the effectiveness of comprehensive, multidisciplinary management, and provides guidelines for optimizing disease management.

A 40-year-old male patient presented with a one-year history of constipation and weight loss. He reported the passage of narrow-calibre stools, approximately the width of a little finger, and a 10-kilogram weight loss over the past year. A diagnosis of WD was indicated by bronchoalveolar lavage (BAL) fluid analysis, but no microbiological evidence of rectal *T. whipplei* infection was found at that time. The patient received 14 days of intravenous antibiotic therapy, followed by a one-year course of oral cotrimoxazole, combined with a comprehensive management plan that included structured medication adherence education, targeted intestinal nursing care, nutritional support, psychological intervention, and multidisciplinary follow-up. After five months of treatment, the patient showed significant improvement, including four-kilogram weight gain, a return to normal bowel movements, and resolution of pulmonary inflammation.

This case highlights the importance of early recognition of atypical WD manifestations and demonstrates that a multidisciplinary model integrating standardized antimicrobial therapy with structured nursing management may significantly improve patient outcomes in rare systemic infections.

## INTRODUCTION

Whipple’s disease (WD) is a chronic, curable systemic infection caused by the gram-positive bacterium *Tropheryma whipplei*. It was first described by Dr. George Hoyt Whipple in 1907 and was confirmed by small intestinal biopsy in 1958.^1^–^3^ The annual incidence of WD is approximately less than one case per million people in the general population; WD affects mainly white patients in middle age (the mean age at the time of diagnosis is 55 years). Notably, 85% of WD patients are males.^4^ Owing to the disease’s rarity and diverse clinical presentations, the average time from symptom onset to diagnosis often exceeds six years.^5^ Classic WD generally manifests as intermittent arthralgia, arthritis, or other joint discomfort accompanied by chronic digestive symptoms (diarrhea and abdominal pain).^6^,^7^ Owing to *Tropheryma whipplei* infection, which primarily affects the gastrointestinal tract, approximately 25.5% of WD patients are admitted to hospitals because of gastrointestinal manifestations.^8^ In terms of pathological mechanisms, immune reconstitution inflammatory syndrome (IRIS), characterized by exaggerated and dysregulated inflammatory responses following *Tropheryma whipplei* infection, plays a crucial role in WD progression. Specifically, the gastrointestinal tract exhibited significant reconstitution of nonspecific T-helper 1 (Th1) cells and altered the production of cytokines, including tumor necrosis factor-α (TNF-α) and interferon-γ (IFN-γ). These cytokines cause damage to intestinal epithelial cells and promote intestinal epithelial permeability. 9-10

This study presents a case in which a patient was diagnosed with WD via bronchoalveolar lavage (BAL) secondary to rectal stenosis. Notably, no evidence of Tropheryma whipplei infection was found in the rectum, and the patient’s malnutrition and stenosis symptoms improved after treatment.

## CASE REPORT

### Chief complaint:

A 40-year-old male patient presented with a one-year history of dyschezia.

### Disease history:

He denied nausea, anorexia, or vomiting. His stools were narrow, frequent (approximately 4–5 times daily), and low in volume. Starting from August 2022, his dyschezia worsened, with bowel movements occurring only once every 5–7 days and a significant reduction in the volume of the stool. The patient subsequently visited a local oncology hospital. To rule out malignancy, ultrasound-guided needle biopsy and PET-CT scans were performed, both of which yielded negative results. However, his weight had decreased from 60 kg to 50 kg over the past year. Owing to persistent concerns, the patient was referred to Hangzhou Third People’s Hospital for further evaluation and treatment. Colonoscopy revealed annular rectal stenosis with perimucosal edema that persisted despite fasting, an enema, and osmotic laxatives (Fig.1).

**Fig.1:**
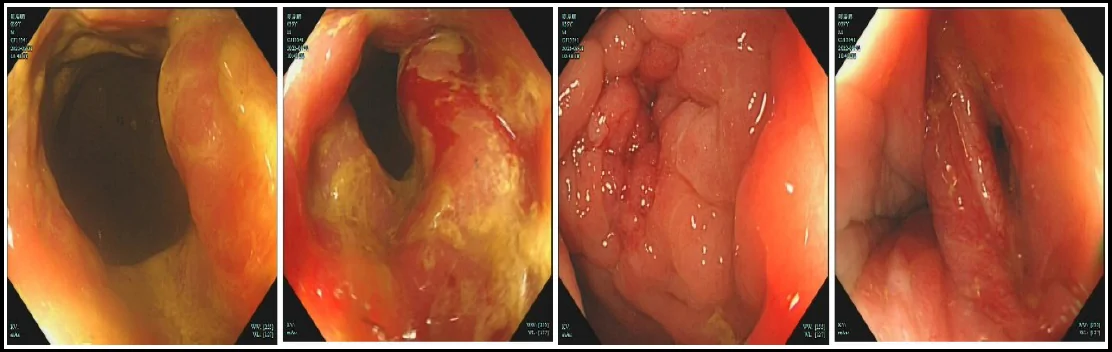
Colonoscopy (pretreatment) showing annular rectal stenosis and mucosal edema of the surrounding bowel wall.

### Ethical approval:

The ethics committee of our hospital approved this study (No. 2026KA067; Date: 2026-02-13), and written informed consent was obtained.

### Exams

Postprocedure pathology indicated chronic inflammation. The results of gastroscopy were unremarkable, and the results of routine blood tests, blood biochemistry tests, and tumor marker tests were all within normal limits. Fecal occult blood and leukocyte tests were positive. A CT scan (August 23, 2022) revealed inflammation in the right middle lobe of the lung (Fig.2). Bronchoalveolar lavage (BAL) revealed Tropheryma whipplei DNA, prompting the respiratory team to recommend treatment for *T. whipplei* infection. However, next-generation sequencing (NGS) of colon tissue was negative for *T. whipplei*. Despite a negative colonic NGS result, the patient was treated for suspected *T. whipplei* pneumonia with intravenous meropenem (1 g every eight hours) for 14 days.

**Fig.2:**
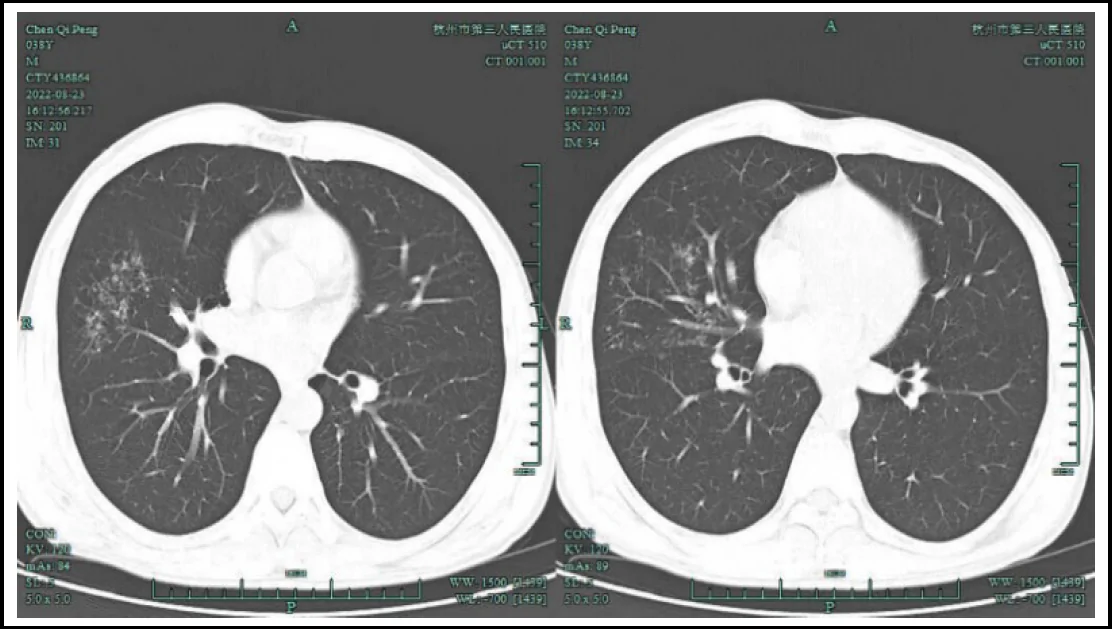
Chest CT scan (August 23, 2022) showing pulmonary inflammation in the right middle lobe.

### Diagnosis: WD

### Treatment

Symptom treatment was administered for abdominal distension and dyschezia. Upon discharge, the patient was advised to continue antibiotic therapy with sulfamethoxazole (SMZ)-trimethoprim (TMP) tablets (each tablet containing 0.4 g SMZ and 80 mg TMP) at a dosage of two tablets twice daily for one year to consolidate the treatment of pneumonia.

In addition to medical and endoscopic treatment, a structured nursing management plan was implemented throughout hospitalization and follow-up. The nursing interventions included the following:

### Medication adherence management:

Given the patient’s intermittent adherence to SMZ therapy due to memory-related difficulties, individualized medication management strategies were implemented. These included using pill organizers and alarm-based reminders and involving family members to assist with supervision. Health education was provided to specify the prescribed dosage (two tablets twice daily) and emphasize the importance of completing the one-year antimicrobial regimen to prevent recurrence. Telephone follow-up was scheduled every two weeks to monitor medication compliance and reinforce adherence strategies.^11^

### Targeted Intestinal Nursing Care:

Based on the presence of annular rectal stenosis, dyschezia, positive fecal occult blood, and fecal leukocytes, symptom-oriented bowel management was initiated. Upon admission, fasting, enemas, and prescribed laxatives were administered to reduce bowel wall edema and relieve obstruction-related symptoms. Postprocedure nursing care included systematic monitoring of stool frequency, caliber, volume, abdominal distension, and signs of gastrointestinal bleeding. Observational data were documented to assess intestinal function and mucosal recovery.^12^

Considering the coexistence of pulmonary inflammation and intestinal lesions, nursing care was coordinated with the respiratory and gastroenterology teams. Imaging examinations and routine laboratory tests were arranged as clinically indicated to guide ongoing management.^13^ Follow-up was conducted through scheduled outpatient visits and telephone monitoring to assess symptom progression, treatment adherence, and potential complications.

A structured dietary plan was developed to support weight recovery and reduce intestinal burden. The patient was advised to consume high-protein, high-fiber, and easily digestible foods while avoiding spicy and hard-textured foods to minimize mucosal irritation. Adequate fluid intake was encouraged to facilitate bowel movements. Body weight was monitored regularly in accordance with nutritional nursing recommendations.^12^

Following endoscopic balloon dilation, the patient and family members received instructions on gentle manual anal dilation exercises lasting 5–10 minutes per session.^14^ Ongoing monitoring included evaluation of stool characteristics, frequency, abdominal symptoms, and signs of hematochezia, with comparisons to baseline measurements to assess progression.^15^

Targeted psychological support was provided to address the emotional distress associated with prolonged diagnostic uncertainty and treatment burden. Education was provided regarding disease progression, treatment objectives, and the importance of adherence. Structured, simplified nursing instructions were implemented to improve patient confidence and compliance.^15^

### Follow-up:

The follow-up plans included chest CT and colonoscopy at six months to assess progression. At the five-month follow-up visit, the patient had not adhered to the prescribed one-year SMZ regimen and had only taken the medication regularly for three months. Despite this nonadherence, he reported significant improvement in dyschezia, with increased stool caliber and a normalized diet. Furthermore, he had no abdominal distension and had gained nearly 4 kg of weight. A follow-up chest CT scan on February 6, 2023 (Fig.3), revealed partial improvement with reduced evidence of pulmonary inflammation. Colonoscopy revealed persistent rectal stenosis, which improved compared with baseline. The colonic mucosa also improved, with reduced edema (Fig.4). Suspecting that the rectal stenosis was a fibrotic stricture secondary to chronic infection, endoscopic balloon dilation was recommended. The patient recovered well postoperatively and was discharged three days after the endoscopic procedure was successful. He was instructed to continue SMZ and return for a second endoscopic balloon dilation procedure within three months. A follow-up phone call one month after discharge revealed that the patient refused the recommended second dilation because they felt well. In addition, the patient acknowledged intermittent SMZ use because of memory issues, despite the persistence of stenosis.

**Fig.3:**
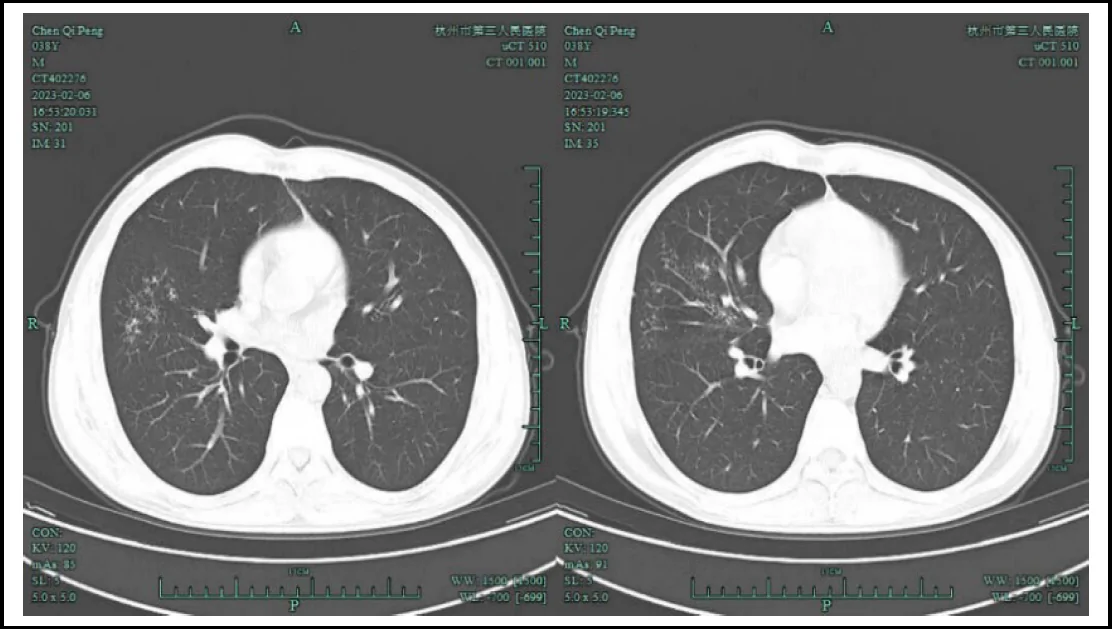
Chest CT scan (February 6, 2023) showing partial resolution of pulmonary inflammation following treatment.

**Fig.4:**
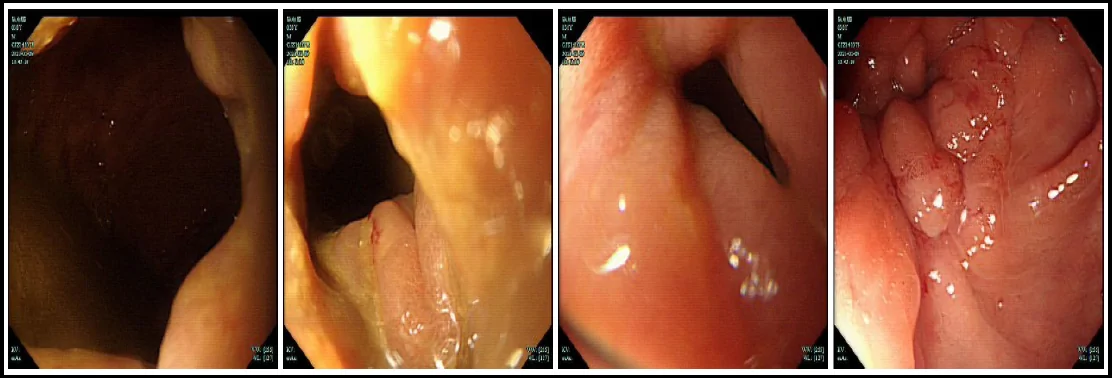
Colonoscopy (February 6, 2023) showing persistent but improved rectal stenosis with reduced mucosal edema.

## DISCUSSION

This study reports the case of a 40-year-old male patient with WD complicated with rectal stenosis. The main symptoms were long-term difficulty in defecation, thinning of feces, and a significant decrease in body weight. The diagnosis of WD was suggested by PCR testing of bronchoalveolar lavage fluid, and standardized intravenous combined oral antibacterial treatment was given. Under a multidisciplinary nursing management strategy, multidisciplinary comprehensive management was implemented, and the patient’s body weight improved, defecation function improved, rectal stenosis eased, and lung inflammation significantly decreased. The clinical outcome was good. On the basis of existing research results at home and abroad, a comprehensive analysis of the clinical characteristics, diagnosis, and treatment process, nursing effects, and similarities and differences in the literature of this case is conducted to provide a basis for the identification and management of rare diseases in clinical practice.

Owing to the diverse manifestations of WD, it is easily misdiagnosed and missed in clinical practice.^14^,^15^ Research has shown that the overall incidence rate of WD is less than one in a million, and it tends to occur in Caucasian men aged approximately 55 years, with a male to female ratio of 8~9:1.^16^,^17^ The MHC - II/HLA alleles DRB113 and DQB106 are important genetic predisposing factors.^14^,^18^ Pathogenic bacteria are transmitted mainly through contaminated water sources, with infection rates ranging from 2% to 11% among the general population, while high-risk occupations and special populations have relatively high infection rates.^19^–^21^ Tropheryma whipplei is a Gram-positive bacterium closely related to Actinobacteria. After infection, it can cause symptoms such as lymphatic stasis, nutrient absorption disorders, and chronic diarrhea.^11^,^13^,^14^ This patient presented with decreased body mass, intestinal symptoms, and pulmonary involvement, which is consistent with the clinical features of classical WD. However, rectal stenosis was observed during the course of the disease. After a search of domestic and foreign literature revealed that there are currently no reports of rectal stenosis caused by WD, which is the most important clinical discovery of this study.

Classic WD often presents with digestive tract involvement as the core manifestation, gradually leading to abdominal pain, diarrhea, fatty diarrhea, joint pain, and severe malnutrition after prodromal symptoms. The small intestine is the most affected area, and some patients may experience colitis, mesenteric lymph node enlargement, and even gastrointestinal bleeding.^22^,^23^ At present, abnormal host immune function is believed to be a key mechanism for the occurrence and development of diseases. Defects in macrophage function and a weakened type 1 T-cell response can lead to the sustained colonization of pathogens, chronic inflammation that is difficult to control, and ultimately the progression to systemic lesions.^24^–^26^

Notably, the majority of infected individuals are asymptomatic carriers, and only a few individuals develop overt disease due to immune imbalance, indicating that immune status plays a decisive role in disease progression.^27^ The diagnosis of WD relies on clinical symptoms, histopathology, and molecular biology testing. PAS staining can reveal pathogenic bacterial inclusions, but its specificity is limited. PCR detection has become the key to diagnosis because of its high sensitivity and specificity. The detection rate of pathogenic bacteria in bronchoalveolar lavage fluid is higher than that in saliva and blood samples.^28^,^29^

Under endoscopy, the duodenal mucosa may exhibit erythema, edema, widened folds, and characteristic yellow–white changes.^14^,^30^ This case was diagnosed on the basis of PCR-positive bronchoalveolar lavage fluid, typical clinical manifestations, and auxiliary examination results, and the diagnostic process met the recommended standards in the literature. Unlike in previous studies, no microbiological evidence of rectal *T. whipplei* infection was found at the initial diagnosis in this case. However, after standardized antibacterial treatment, rectal stenosis improved synchronously with nutritional status and intestinal symptoms, suggesting that persistent intestinal inflammation, mucosal damage, and fibrosis remodeling caused by chronic infection of WD may be involved in the formation of rectal stenosis, providing a new understanding of the scope of gastrointestinal involvement and types of complications in this disease.^31^,^32^

Compared with the literature, the greatest difference in this case is the rare clinical manifestation of WD combined with rectal stenosis. Currently, there are no similar case reports at home or abroad. Most studies focus on the manifestations of WD in the small intestine, joints, nervous system, and lungs, with very little attention given to the low intestine, especially in terms of rectal structural damage.^33^–^36^ After the full treatment course of antibacterial and comprehensive nursing intervention, the difficulty of defecation was relieved, the shape of the feces was restored, and the degree of rectal stenosis was reduced in this case, further supporting the potential association between infection, inflammation, mucosal injury, and stenosis formation. This case provides the first reported clinical observation of WD involving the rectum and causing stenosis, enriching the clinical phenotype spectrum of the disease and improving clinical awareness of rare gastrointestinal complications. It has important value in the literature and clinical guidance.

The antibiotic regimen resulted in rapid improvement in WD during the early phase. The first effective treatment occurred in 1952 with chloramphenicol.^37^ Numerous methods have been documented for treating *T. whipplei* infections. Initially, during the 1960s, penicillin and streptomycin were administered intravenously for two weeks, followed by oral tetracycline for three to 14 months.^38^ In the 1980s, for cases involving the central nervous system, trimethoprim and sulfamethoxazole, which are capable of crossing the blood–brain barrier, were used, although relapses were possible.^39^-^40^ Later, in the 1990s and 2000s, the treatment typically involved intravenous streptomycin and penicillin or ceftriaxone for two weeks, followed by oral trimethoprim with sulfamethoxazole for 1-2 years.^41^-^42^ However, the use of trimethoprim with sulfamethoxazole is debated, as *T. whipplei* is not susceptible to trimethoprim, and there are concerns about sulfamethoxazole resistance.^43^ In addition, SMZ tablet administration has shown common side effects, including nausea, vomiting, and allergic reactions, which might cause poor adherence in patients with WD.

In terms of nursing management, this study focuses on multidisciplinary nursing management and involves collaboration with a multidisciplinary team to implement comprehensive and individualized interventions, including medication adherence management, targeted intestinal care, collaborative follow-up, nutritional support, manual anal dilation, and psychological intervention. The results revealed that through medication box packaging, regular reminders, family supervision, and health education, patients’ medication compliance can be significantly improved, and inflammation recurrence caused by self-discontinuation of medication can be avoided. Through targeted intestinal care, anal dilation training, and dietary guidance, it is possible to effectively reduce intestinal mucosal edema, improve bowel movements, and promote mucosal repair.

Multidisciplinary collaboration and regular follow-up can dynamically monitor changes in condition, adjust intervention strategies promptly, and ensure the continuity of treatment. The above nursing measures are closely related to improvements in clinical outcomes, reflecting the application value of multidisciplinary nursing management in rare and complex cases. At present, few studies have integrated systematic nursing pathways into the comprehensive management of Whipple’s disease. The “anti-infection treatment plus multidisciplinary nursing management plus multidisciplinary collaboration plus long-term follow-up” model constructed in this study provides a replicable and scalable nursing plan for patients with similar rare diseases.

### Strengths

This study reported a rare case of WD complicated with rectal stenosis, clarified its clinical relevance, and filled the literature gap in the relevant field; the diagnostic standards, complete treatment, and nursing measures system formed a complete chain of evidence for diagnosis, treatment, and nursing care; and multidisciplinary nursing management concepts were combined to carry out multidisciplinary management and provide a practical reference for the individualized care of rare diseases. The follow-up data are complete, the clinical process is clear, and the credibility of the evidence is high.

### Limitations

First, this is a single case report with a limited sample size, which makes determining the exact incidence and independent risk factors for rectal stenosis caused by Whipple’s disease difficult. Second, analyses of intestinal mucosal inflammatory factors, fibrosis pathways, and microbial community structure have not been carried out, and the molecular mechanism of stenosis formation still needs further verification. Finally, owing to the limited follow-up time, the risk of long-term recurrence, degree of intestinal function recovery, and long-term prognosis of patients still need to be continuously evaluated.

In summary, the clinical occurrence of Whipple’s disease combined with rectal stenosis is extremely rare and easily overlooked and misdiagnosed. This study suggests a possible manifestation of this complication, and under the guidance of standardized antibacterial treatment combined with multidisciplinary comprehensive management, it can significantly improve patients’ symptoms, body mass, and intestinal function, with definite clinical effects. This case provides new evidence for the rare phenotype of Whipple’s disease worldwide and provides important references for clinical diagnosis, nursing practice, and mechanism research. It has significant medical value and clinical significance.

## CONCLUSIONS

WD complicated with rectal stenosis is clinically rare and prone to misdiagnosis in the early stages. In this case study, the diagnosis was suggested by PCR on bronchoalveolar lavage fluid (BALF). Following standardized intravenous-to-oral antimicrobial therapy combined with endoscopic balloon dilation, manual anal dilation, and multidisciplinary follow-up care, the patient achieved improvement in dyschezia, weight gain, and resolution of pulmonary inflammation. These outcomes suggest that a comprehensive management model that combines adequate dosing, full-course antimicrobial therapy, targeted intestinal care, adherence management, nutritional guidance, and psychological support was associated with favorable clinical outcomes in this case. Future studies with larger sample sizes and prospective designs are needed to further validate the safety and long-term efficacy of this nursing pathway.

### Future perspectives:

Multicenter retrospective studies should be conducted to systematically summarize the rare and uncommon clinical manifestations of Whipple’s disease to improve early diagnosis rates; in-depth exploration of the pathophysiological mechanisms of chronic infection, inflammation activation, intestinal wall fibrosis, and intestinal stenosis should be performed; standardized diagnosis, treatment, and nursing processes for Whipple’s disease and rare complications should be established; and multidisciplinary collaboration models should be optimized to improve long-term patient outcomes.

### Authors’ Contributions:

**FD:** Literature search, study design and manuscript writing.

**LD, YH, WW** and **RX:** Data collection, data analysis and interpretation. Critical review.

**FD:** Manuscript revision and validation and is responsible for the integrity of the study.

All the authors have read and approved the final manuscript.
